# Using the Wii Fit as a tool for balance assessment and neurorehabilitation: the first half decade of “Wii-search”

**DOI:** 10.1186/1743-0003-11-12

**Published:** 2014-02-08

**Authors:** Daniel J Goble, Brian L Cone, Brett W Fling

**Affiliations:** 1 Sensory-Motor and Rehabilitative Technology Laboratory (SMaRTlab), School of Exercise and Nutritional Sciences, San Diego State University, 5500 Campanile Drive, San Diego, CA 92182-7251, USA; 2 Balance Disorders Laboratory, Department of Neurology, Oregon Health and Science University, 505 NW 185th Avenue, Beaverton, OR 97006, USA

**Keywords:** Postural control, Interventions, Training, Virtual reality, Evaluation

## Abstract

The Nintendo Wii Fit was released just over five years ago as a means of improving basic fitness and overall well-being. Despite this broad mission, the Wii Fit has generated specific interest in the domain of neurorehabilitation as a biobehavioral measurement and training device for balance ability. Growing interest in Wii Fit technology is likely due to the ubiquitous nature of poor balance and catastrophic falls, which are commonly seen in older adults and various disability conditions. The present review provides the first comprehensive summary of Wii Fit balance research, giving specific insight into the system’s use for the assessment and training of balance. Overall, at the time of the fifth anniversary, work in the field showed that custom applications using the Wii Balance Board as a proxy for a force platform have great promise as a low cost and portable way to assess balance. On the other hand, use of Wii Fit software-based balance metrics has been far less effective in determining balance status. As an intervention tool, positive balance outcomes have typically been obtained using Wii Fit balance games, advocating their use for neurorehabilitative training. Despite this, limited sample sizes and few randomized control designs indicate that research regarding use of the Wii Fit system for balance intervention remains subject to improvement. Future work aimed at conducting studies with larger scale randomized control designs and a greater mechanistic focus is recommended to further advance the efficacy of this impactful neurorehabilitation tool.

## Introduction

On December 1^st^, 2012 the Nintendo Wii Fit gaming system celebrated its fifth anniversary. This novel system consists of exercise-based game software, the Wii console, and a specially developed Wii Balance Board (WBB) controller. According to Nintendo, the purpose for developing the Wii Fit was to combine fun and fitness for all ages, including such aspects of physical well-being as joint flexibility, muscle strength, and upright standing posture. The system is relatively inexpensive (~$200 US), easy to access, and the games are highly engaging [1-3]. Based on these characteristics, it is not surprising that the Wii Fit software is one of the top selling video console games of all time.

**Figure 1:**
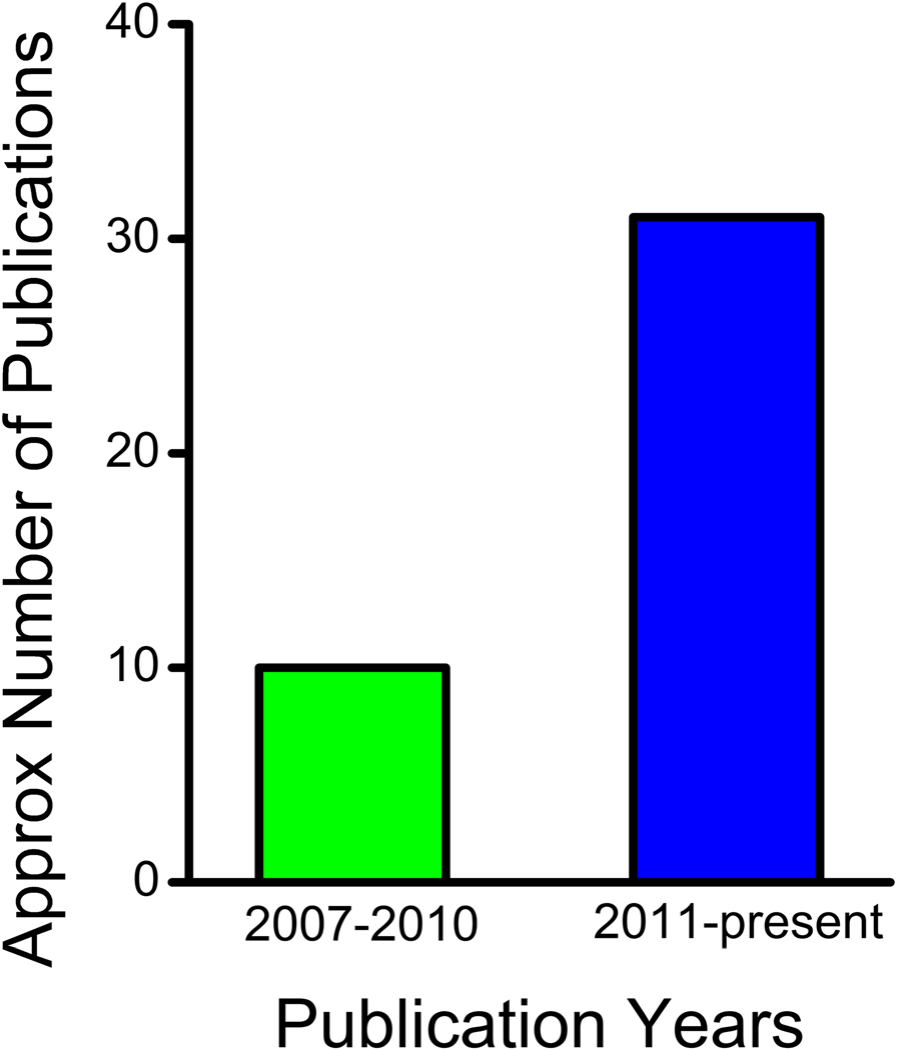
**Growth in Wii Fit Research.** From 2011 till the fifth anniversary the number of Wii Fit-balance related papers has shown an exponential increase [1,2,4-42].

Beyond its use in the general population, the Wii Fit has generated significant interest in the neurorehabilitation research domain. This is particularly true with respect to the study of balance control, where the Wii Fit software and/or WBB have provided an increasingly attractive means of assessing and training individual balance ability. The number of balance-related research papers using the Wii Fit system (Figure 1) tripled in the two years prior to the fifth anniversary, compared the first three years of its existence [1,2,4-42]. Undoubtedly, balance research using the Wii Fit is gaining in both popularity and acceptance within the scientific community.

The ubiquitous importance of balance research for neurorehabilitation is unquestioned based on epidemiological reports indicating that older adults and various individuals with neuromuscular disability conditions have an increased likelihood for catastrophic fall-related injuries [43-47]. The cost of such injures is not just to the individual but to the greater health care system at large. To this extent, the goal of the present review was to summarize the first half decade of Wii Fit balance research and provide insight regarding the important results that will shape the next five years. Literature for this review was obtained through a targeted search of PubMED and MEDline databases using keywords such as “Wii” and “Balance”. Papers were included that used the Wii Fit system or WBB for the assessment or training of balance ability. In the first half of this review, approaches are overviewed that utilized the Wii Fit as a neuroengineering tool for balance assessment. Alternatively, the second half of this review discusses detailed evidence supporting use of the Wii Fit as a balance intervention tool for both healthy and diseased populations.

### Balance assessment using the Wii Fit system

#### Comparing the WBB and a scientific grade force platform

Perhaps the most significant neuroengineering-based innovation to arise with the advent of the Wii Fit gaming system was the creation of the WBB itself. This device, while limited to some extent in terms of weight capacity (~150 Kg) and sampling frequency (~30-50 Hz), can be utilized as a cost-efficient and transportable version of a scientific-grade force platform. Force platforms are currently the gold standard for assessment of balance ability in many laboratories and clinics, but cost between $5000-20000 US. These devices provide accurate measures of body center of pressure (COP), which is an approximation of the body’s center of mass (or balance point) projected vertically onto the floor below. COP is an important metric for balance stability assessment given that it closely approximates body sway.

When standing on the WBB, the Wii Fit system measures body sway in the side to side (COPx) and front to back (COPy) directions based on downward force sensor data generated at each corner of the WBB according to the following equations:

$$\text{COPx}=21*\left(\left(\text{TR}+\text{BR}\right)‒\left(\text{TL}+\text{BL}\right)\right)/\left(\text{TL}+\text{TR}+\text{BL}+\text{BR}\right)$$

$$\text{COPy}=12*\left(\left(\text{TL}+\text{TR}\right)‒\left(\text{BL}+\text{BR}\right)\right)/\left(\text{TL}+\text{TR}+\text{BL}+\text{BR}\right)$$

In these equations TR, TL, BR and BL are the force sensor values from the top right, top left, bottom right and bottom left corners of the WBB. This information, along with total downward force (i.e. vertical ground reaction force), is transmitted with minimal time lag to the Wii console via wireless (i.e. Bluetooth) technology.

In addition to communicating with the Wii console for gaming applications, WBB wireless signals are also readily available for use with other Bluetooth compatible devices such as personal computers and laptops. To this extent, once synced to a device, it is possible to create customized WBB software applications in a number of computer programming environments (e.g. LabVIEW, MATlab,) based on COP data and/or the total downward force obtained directly from the WBB in real-time. This straightforward means of unlocking the Wii has opened the door for the development of many custom balance applications.

**Figure 2:**
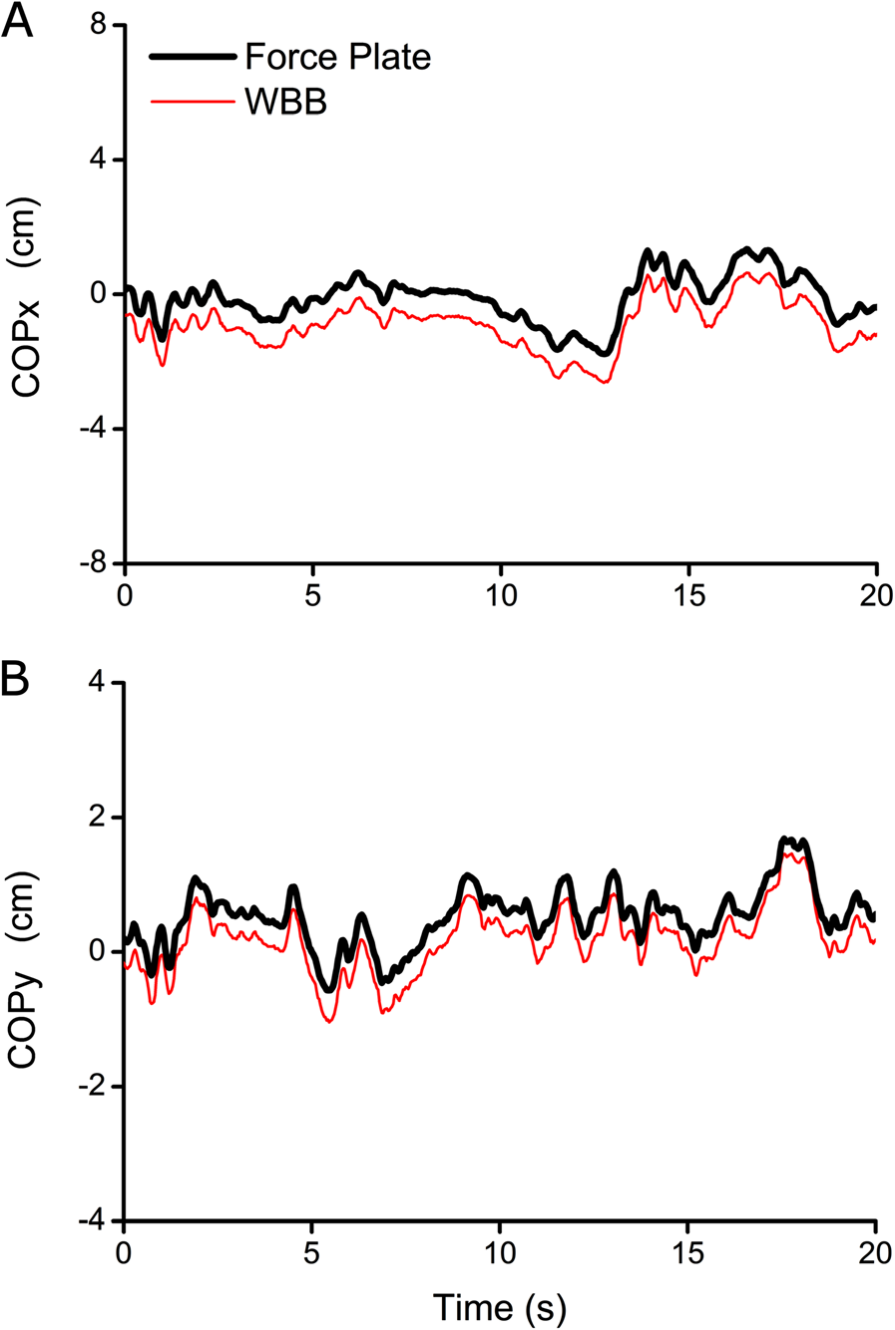
**Representative COPx (A) and COPy (B) data for the force plate and WBB over the course of an example 20 s static balance trial.** Pearson Correlation between the two signals is r = 0.99.

Using the WBB and custom software, Clark and colleagues [11] performed an initial, and critical, validation study of COP information outputted from a WBB. In this study, the total length of COP traces was compared between a WBB and scientific grade force plate during ten, 30s trails of standing with eyes opened/closed in single or double stance conditions. The results showed that COP path lengths obtained from the WBB were valid, correlating well with those from the scientific grade force platform. The COP data were also reliable, evidenced by strong interclass correlations between trials of similar balance conditions. These findings have since been replicated in healthy adults by Hubbard and colleagues [26], as well as by Holmes et al. [39] for assessment of postural disturbances in Parkinson’s disease. Sample data from our own lab is shown in Figure 2, comparing 20 s standing on a WBB and a sophisticated force plate (model: OR6-7-2000, Advanced Mechanical Technology, Inc., Watertown, MA, USA). Taken together, it can be concluded that the WBB provides COP data that is both valid and reliable. This information can subsequently serve as a cost effective proxy for force plate COP in the development of portable posturography systems.

#### Examples of balance assessment using custom WBB COP data applications

Several attempts have been made to use raw COP data from a customized WBB for balance assessment. Young et al. [19], demonstrated that customized WBB COP data could be used to assess changes in older adult balance following a specially designed, game-based intervention over four weeks. Before and after training, six elderly participants undertook a traditional balance test (i.e. Romberg Test) whereby they stood as still as possible on the WBB for two, 30 s trials - one with eyes open and one with eyes closed. The COP data collected showed anterior-posterior COP variability was positively reduced by the intervention, but only in more difficult trials where visual information was not available (i.e. eyes closed conditions). Increases in body sway for individuals with Autism [12] and Hearing Loss [37] have also been measured with custom WBB COP.

In contrast to the traditional balance assessment utilized by Young et al. [19], Clark et al. [16] described a slightly more novel method for assessing an important aspect of balance – weight bearing asymmetry. Weight bearing asymmetry is prevalent in many clinical populations, whereby greater body weight is placed on, typically, the less impaired limb. In this study, 23 healthy young adults stood with each leg on a different WBB, and performed five squats with and without visual feedback regarding weight bearing asymmetry. The results showed that participants could reduce asymmetry when visual feedback was provided. In future work, this method could be utilized to enhance known weight bearing asymmetries in individuals with hemiplegia due to, for example, stroke or cerebral palsy. Further, these findings provide an excellent example of the potential for creating novel balance tools with WBB technology. For other examples of non-traditional COP-based applications of the WBB see Kennedy et al. [22] and Yamamoto et al. [41].

#### Balance metrics provided by the Wii Fit game software

The Wii Fit software includes several means of assessing balance ability. Once the Wii Fit software is loaded, participants are first encouraged to perform a “body test” that comprises both “center of balance” and “body control” measures of balance control. In the center of balance assessment, participants stand still on the WBB for 7 s while COP data is collected. From this data, the participant’s average COP deviation from body midline is calculated, indicating a measure of weight bearing asymmetry. Overall, this assessment is thought to provide information about body posture with the assumption that having an equal distribution of weight between left and right legs is ideal.

The second balance metric garnered by the Wii Fit software is based on a body control test. Unlike the center of balance test, the body control test is not an assessment of static COP but, rather, a complex combination of cognitive and dynamic COP control. Individuals are given one assessment “game” at random from a group of ten possible choices. The games range from simple tests of how accurately individuals can move a cursor representing their COP on the television screen to a target location, to those that are more cognitive in nature (i.e. involving counting, ordering and memorizing of items). Once completed, the participant is given a score called the “Wii Fit age” for their performance. This metric ranges from 2-99 and represents age-normalized performance such that, for example, a score of 46 indicates balance ability equal to that of the average 46 year old.

#### Examples of Wii Fit software-based balance assessments

Only a few attempts have been made to utilize Wii Fit software-based balance metrics in research settings, and only limited success has been achieved. First, in a study by Dougherty et al. [23], 9 older adults were asked to undergo a 5-week balance training program using a multi-axis balance training board (Indo Board Balance Trainer, EABO Inc.). The participant’s Wii Fit Age was calculated before and after the intervention and results showed that Wii Fit Age was lower (i.e. improved) following training. Despite this mean improvement, however, no statistical differences were found, suggesting that only some of the balance improvement was captured by the Wii Fit Age metric.

To what extent two Wii Fit body test metrics correlate with standardized tests of older adult fitness, balance and mobility was recently determined by Reed-Jones and colleagues [13]. These researchers compared a battery of clinical examinations with the “Basic Balance Test” and the “Prediction test” of the Wii Fit software. Overall, there was little correlation between the clinical tests and the data provided from the Wii Fit software-based tests of balance. This finding mirrored that which was described by Gras et al. [6], who also found that the center of balance test was not a valid measure compared to its counterpart determined via a force platform. Further, Wikstrom [40] found poor validity and reliability for 12 of the exer-games associated with the Wii Fit software compared to a standard balance test. This result may reflect the different requirements of balance for gaming and the balance test utilized by Wikstrom [40]. Taken together, however, these results suggest that Wii Fit software-based measures of balance ability are not effective.

### Using the Wii Fit for balance intervention

The most prominent scientific use for the Wii Fit has been neurorehabilitation-based balance interventions [1,2,4,7,10,14,15,17-19,21,22,27-31,34,36]. An overview of studies where the balance abilities of healthy adults have been trained is provided in Table 1. This table shows a clear bias toward work with older individuals. In Table 2, those studies are outlined that have used the Wii Fit system as a means for neurorehabilitating poor balance in various clinical conditions. This work has focused on a number of clinical populations including Stroke, Parkinson’s Disease and Cerebral Palsy. Specific detail regarding the intervention types utilized, and their efficacies are provided herein.

**Table 1:** Wii Fit intervention studies that trained balance in healthy adults

**Author(s)**	**Intervention details**	**Population**	**Balance-specific**	**Statistical**
		**(Group size)**	**Outcome measure**	**Significance?**
Bateni (2012) [36]	Ski Slalom, Ski Jump and Table	Older	Berg balance scale	Not tested
	Tilt Wii Fit games	Adults (n = 5)	Bubble balance score	Not tested
	Dose: 12, 20 min sessions			
Padala et al. (2012) [34]	Various Wii Fit games selected	Older	Berg balance scale	p < 0.01
	from all categories	Adults (n = 11)	Tinetti test	p < 0.05
	Dose: 40, 30 min sessions		TUG (normal) p > 0.05	p>0.05
Toulotte et al. (2012) [14]	Various Wii Fit games selected	Older	Unilateral stance test	p > 0.05
	mostly from yoga a balance types	Adults (n = 9)	Tinetti test	p < 0.05
	Dose: 20, 60 min sessions		Wii fit center of balance	p < 0.05
Rendon et al. (2012) [30]	3 Wii Fit games – not specified	Older	TUG (normal)	p < 0.05
	Dose: 18, 40 min sessions	Adults (n = 16)	ABC	p < 0.05
Franco et al. (2012) [28]	Soccer heading, Ski jump, Ski	Older	Berg balance scale	p < 0.001
	slalom, Tightrope and Table tilt	Adults (n = 11)	Tinetti test	p < 0.01
	Wii Fit games			
	Dose: 6, 15 min sessions			
Young et al. (2011) [19]	Two custom-designed games	Older	ML/AP COP variability eyes open	p > 0.05
	controlled by WBB	Adults (n = 6)	ML COP variability eyes closed	p > 0.05
	Dose: 10, 20 min sessions		AP COP variability eyes closed	**p < 0.05**
			Tinetti falls efficacy scale	Not given
Williams et al. (2011) [15]	Self-selected Wii Fit games from	Older	Berg balance scale	**p < 0.01**
	balance and aerobics categories	Adults (n = 22)		
	Dose: 12, 20 min sessions			
Agmon et al. (2011) [18]	Basic step, Soccer heading, ski	Older	Berg balance scale	**p < 0.05**
	slalom and Table tilt Wii Fit games	Adults (n = 7)		
	Dose: 50, 30 min sessions			
Nitz et al. (2010) [7]	Self-selected Wii Fit games from	Adults (n = 8)	TUG (normal and cognitive)	p > 0.05
	all categories		mCTSIB (foam, eyes closed)	p > 0.05
	Dose: 20, 30 min sessions		mCTSIB (unilateral, eyes closed)	**p < 0.05**
			Limits of stability test	p > 0.05
Pigford et al. (2010) [1]	Ski slalom, Table tilt and Deep	Older	Berg balance scale	Not tested
	breathing Wii Fit games	Adults (n = 1)	TUG (normal)	Not tested
	Dose: 10, 20 min sessions		ABC	Not tested
Hanneton and Hanneton (2009) [2]	Deep breathing, Warrior posture	Adults (n = 4)	Improvement in game scores	Not tested
	Torso Twist, Soccer heading, Ski			Not tested
	slalom, Ski jump, Table tilt and			
	Basic step games Wii Fit games			
	Dose: 10, 37 min sessions			

*Abbreviations: ABC* activities-based balance confidence scale, *MCTSIB* modified Clinical Test for Sensory Integration of Balance, *ML* Medial/Lateral, *AP* Anterior/Posterior.

**Table 2:** Wii Fit Intervention studies that rehabilitated balance in clinical populations

**Author(s)**	**Intervention details**	**Population**	**Balance-specific**	**Statistical**
			**Outcome measure**	**Significance?**
Nilsagard et al. (2012) [29]	Perfect 10, Ski/snowboard	Multiple	TUG (normal and cognitive)	p < 0.05
	slalom, Table tilt(+), Penguin	Sclerosis (n = 41)	Four square step test	p < 0.01
	slide, Soccer heading, Tightrope,		ABC	p < 0.05
	Skateboard and balance			
	bubble(+)Wii Fit games			
	Dose: 12, 30 min sessions			
Esculier et al. (2012) [31]	Table tilt, Ski slalom, Balance	Parkinson’s	One-leg stance duration	p < 0.001
	bubble, ski jump and penguin	Disease (n = 10)	COP velocity variability	p < 0.05
	slide Wii Fit games		ABC	p > 0.05
	Dose: 18. 40 min sessions		TUG (normal)	p < 0.05
			Tinetti’s test	p < 0.05
			CBM	p < 0.001
Miller et al. (2012) [27]	Wii Fit games selected based on	Transfemoral	Biodex balance test	Not tested
	participant preference	Amputation (n = 2)	ABC	Not tested
	Dose: 12, 20 min sessions			
Kennedy et al. (2011) [22]	Custom designed “WiiHab” tasks	Acquired	Improvement on custom	Not tested
	using WBB including Sit to Stand,	Brain	designed tasks	Not tested
	Weight Shifting and Stepping	Injury (n = 1)		
	Dose: 6, 12 min sessions			
Zettergren et al. (2011) [17]	Sun salutation, Half moon, Rowing	Parkinson’s	TUG (normal)	Not tested
	squat, Torso twists, Chair, Penguin	Disease (n = 1)	Berg balance scale	Not tested
	slide, Table tilt and Bubble balance			
	Wii Fit games			
	Dose: 12, 50 min sessions			
Gil Gomez et al. (2011) [21]	Custom designed balance games	Acquired	Berg balance scale	p < 0.001
	using WBB including Simon,	Brain	Brunel balance assessment	p < 0.05
	Balloon breaker and Air hockey	Injury (n = 9)	Anterior reach test	p < 0.01
	Dose: 20, 60 min sessions		TUG (normal)	p < 0.01
Shih et al. (2010) [10]	Change of standing posture	Cerebral	Duration of time in desired	
	detector using the WBB	Palsy (n = 2)	standing posture	p < 0.01
	Dose: 45, 3 min sessions			
Deutsch et al. (2009) [4]	Ski jump, Ski slalom, Tightrope,	Chronic	ABC	Not tested
	Lunges and Park stroll Wii Fit games	Stroke (n = 2)	TUG (normal and cognitive)	Not tested
	Dose: 12, 60 min sessions			

*Abbreviations: ABC* activities-based balance confidence scale, *TUG* timed up and go, *CBM* community balance and mobility scale.

#### Types of Wii Fit balance intervention

Most balance interventions involving the Wii Fit system have relied on the balance-specific exer-games included with the Wii Fit software. The goal of the Wii Fit balance games is to move a virtual representation of the participant (called a “Mii”) on a television screen via displacement of participant COP over the WBB. In some games, COP control is only required along a single axis (e.g. medial-lateral), making control of the character more simplistic for the user. In contrast, other games entail greater COP control through simultaneous displacement along both medial-lateral and anterior-posterior axes. Most games last between 30s and 3 min and participants are given a game-specific score after playing that is reflective of their overall performance. An excellent analysis of the various games included in the Wii Fit software was recently provided by Deutsch and colleagues [25].

In addition to the balance games included in the Wii Fit software, some studies have used Wii Fit yoga games to improve participant balance. Rather than focusing on dynamic aspects of COP, as is typical for the Wii Fit balance games, yoga games rely on static balance control. Examples of Wii Fit yoga games are the Sun Salutation and Half Moon poses. In these tasks, participants are led by a virtual trainer on screen, who demonstrates a target body position the participant must achieve. Once in that posture, feedback is given to the participant via a point-representation of their COP in real-time. The participant goal is to keep their body (and COP) as still as possible while maintaining the yoga pose.

Beyond the standard games included in the Wii Fit software, a small number of researchers have sought to create their own balance intervention games using COP data from a customized WBB software application [8,19,21]. This approach allows the creation of games that are customized to fit the needs of a specific population, such as elderly adults or individuals with reduced cognitive function. In addition, these applications have the ability to capture in-game performance, which may be advantageous for developing custom performance metrics. To date, the games created have been quite varied, but generally require the participant to shift their COP to move an object towards or around other objects in a virtual environment. Some examples include catching apples in a basket [8,19], moving a golf ball around barriers towards a hole [8], popping rising balloons/bubbles [19,21] and air hockey [21].

Not all Wii fit balance interventions have been game-based. Shih [42], for example, used a unique approach that encouraged individuals with Cerebral Palsy to become more aware of their COP location. In this experiment, a customized WBB setup was used to activate participants’ favorite television programs when standing on the board with their COP centered between the feet. Deviation from this ideal resulted in the television program being turned off. Even though the participants had reduced cognitive abilities, they were able to implicitly learn COP control over multiple trials and keep the television on for longer periods of time than normal. An increased percentage of trials with the desired COP location were also found in retention trials with the television stimulus constantly on.

#### Efficacy of Wii Fit-based interventions

As can be seen in Tables 1 and 2, a plethora of balance outcome measures have been utilized to assess Wii Fit balance intervention efficacy. The most common tests employed pre and post intervention have been clinical measures of balance such as the Berg Balance Scale [48] and Timed up and Go test [49]. These standard, yet somewhat subjective, measures have been chosen as they are 1) commonly employed in clinical settings, 2) relatively straightforward to administer and 3) do not rely on expensive equipment. Alternatively, some researchers have performed more complex assessments of balance ability such as the sensory organization and limits of stability tests of the Neurocom balance manager system. These latter tests involve state of the art balance equipment and respectively measure the extent to which an individual relies on various sources of sensory feedback (e.g. vision, proprioception, vestibular) during quiet stance, and how far one’s COP can be moved in all directions.

**Figure 3:**
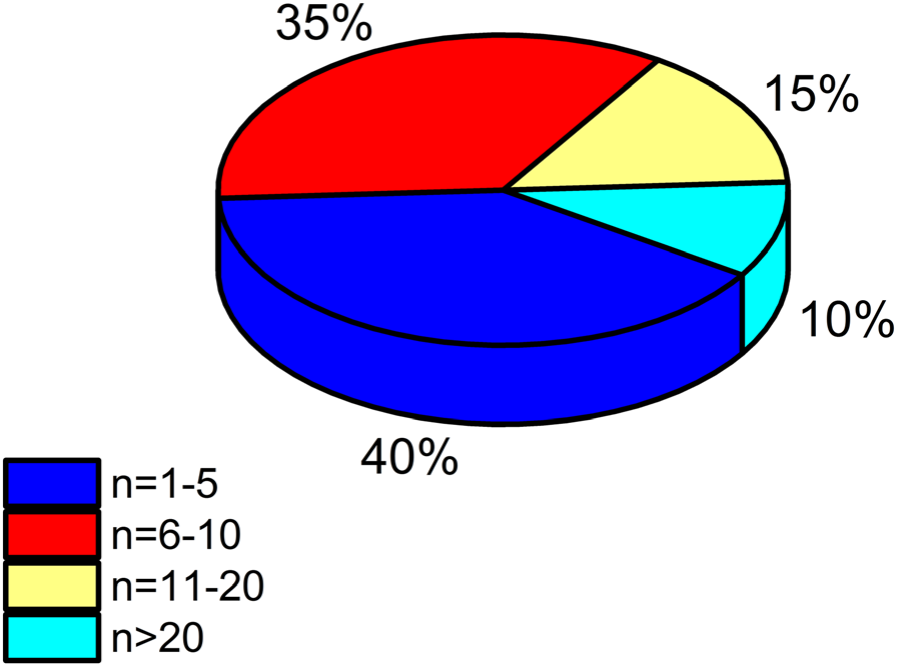
**Group sizes for Wii Fit Interventions.** A majority of Wii Fit intervention studies have utilized group sizes of 10 participants or less. [1,2,4,7,10,14,15,17-19,21,22,27-31,34,36].

Regardless of the outcome measure, Wii Fit interventions have largely been shown to be effective (see Tables 1 and 2). No study has reported a negative impact of Wii Fit training for any measure of balance ability, and most have indicated at least some quantitative or anecdotal evidence of improvement. This is despite a major criticism of the Wii Fit balance work to date - a lack of convincing statistical support. This is likely due to the relatively large number of studies conducted that have utilized small samples of trained individuals. As shown in Figure 3, 40% of studies conducted to date have tested five individuals or less, and 3 out of 4 studies conducted have had 10 participants or less. This underscores the imminent need to go beyond preliminary and case study reports of Wii Fit intervention efficacy and move towards larger-scale, randomized control designs in the future.

Of the studies gathered for this review, only three were identified as having some form of randomized control design. The best insight into the overall effectiveness of Wii Fit interventions might be gained from this work by calculating effect sizes for the mean outcome measure differences. First, in a study by Franco et al. [28], it was shown that six, 15-minute sessions of Wii Fit exer-gaming had a strong effect (Cohen’s d = 0.7) on Berg Balance score in a group of community dwelling older adults compared those that did not use the Wii Fit. Toulotte et al. [14] also found moderate to strong effects across all balance outcome variables in a study of older adults who played Wii Fit games one hour per week for 20 weeks. Balance measures in this study included unilateral stance tests with eyes open (Cohen’s d = 0.5) and eyes closed (Cohen’s d = 1.3), Tinetti’s static (Cohen’s d = 3.8) and dynamic (Cohen’s d = 1.0) tests, as well as the Wii Fit test of center of balance (Cohen’s d = 1.9).

The only randomized control clinical study at the time of the fifth anniversary was carried out by Nilsagard [29] in individuals with multiple sclerosis (MS). MS Patients who played Wii Fit games twice a week for six weeks showed a moderate improvement in the timed up and go test (Cohen’s d = 0.4) and small improvements in the four square step test (Cohen’s d = 0.1) and activities based balance confidence assessment (Cohen’s d = 0.2) compared to a group of individuals with MS that did not play Wii Fit games. These results, while positive, were less impressive than those seen for healthy older adults.

**Figure 4:**
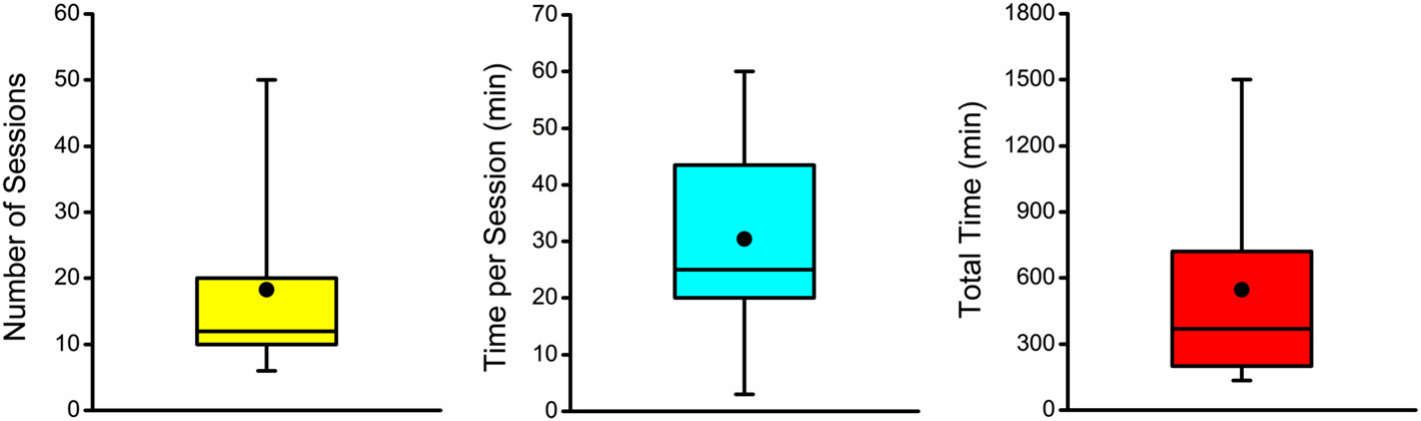
**Wii Fit Intervention Dosing Parameters.** Box and whisker plots representing the 95^th^ percentile (whiskers), interquartile range (box), mean (black circle) and median (black line) data for the various dosing parameters of Wii Fit interventions [1,2,4,7,10,15,17-19,21,22,27-31,34,36].

An important factor influencing the effectiveness of any intervention is the amount of time devoted to the training and rehabilitation tasks (i.e. dosing). In Figure 4, box and whisker plots are given demonstrating the mean, median, inter-quartile range and 95% confidence intervals for the doses associated with Wii Fit interventions. On average, participants have been asked to take part in 19 training sessions with a mean intervention time of approximately 30 min per session. This has resulted in an average of 605 min of training per individual tested. Despite this, there have been several studies with high dose values that appear to skew the mean dosage in the upward direction. In this case, median dose metrics may be more informative of typical intervention characteristics. Using median data, interventions with 12 sessions of 30 min per session would appear to be more normal with an intervention time of 485 min. These values serve as a guideline for optimally designing future Wii Fit interventions. However, additional work focused solely on determining the ideal dosing parameters is still needed to maximize the potential for positive change.

## Summary and conclusions

Use and acceptance of the Wii Fit as a balance assessment and neurorehabilitation tool is growing rapidly. With respect to assessment, initial attempts to use the WBB as a low cost proxy for a scientific force platform via customized software applications appear to be an extremely promising area of exploration. Research relying on measurement of balance ability using Wii Fit software-based metrics, however, has been far less convincing to date. With respect to balance neurorehabilitation, there is reason for optimism regarding the use of Wii Fit-based interventions to foster meaningful balance improvements across a variety of clinical populations. Multiple studies now show improvement in balance control following reasonable periods of training with the Wii Fit games and custom designed applications.

While some evidence has been provided regarding the extent to which training induces shifts in sensory feedback weighting, improvement in sensorimotor processing, or muscle strength gains in association with balance improvements [7], far greater work is needed. In addition, a careful eye should be kept on controlling for various confounds during balance assessment and training protocols. These factors may include participant age, body composition, education level and current medications.

Overall, use of the Wii Fit for assessment and neurorehabilitation purposes is likely to continue to grow in the years to come. Greater emphasis must still be placed on implementing more powerful, randomized control designs with larger sample populations to test questions of interest. There must also be a greater emphasis placed on determining what specific neural and musculoskeletal adaptations occur in response to Wii Fit training, and how those changes compare to other commonly utilized balance training interventions such as uneven balance boards and yoga. Such studies, in association with the information gathered in this review, will form the basis of the next half decade of “Wii-search”.

## Abbreviations

WBB: Wii Balance Board; COP: Center of pressure.

## Competing interests

The authors declare that they have no competing interests.

## Authors’ contributions

DG, BC and BF were all involved in the conception of this work, as well as in the gathering of relevant published research papers on the topic, their synthesis and drafting the manuscript. All authors read and approved the final manuscript.

## Authors’ information

DG is currently an Assistant Professor and director of the Sensorimotor and Rehabilitation Technology Laboratory (SMaRTlab). He has a Master’s degree in Biomechanics, Phd in Human Motor Control and has done post-doctoral work in the field of sensorimotor neuroscience. His past work on balance has focused on proprioception and brain function using neuroimaging.

BC is currently pursuing a Master’s degree in Rehabilitation Sciences and has Undergraduate Training in the field of Exercise and Nutritional Sciences. He has clinical experience in the field of physical therapy including balance intervention for individuals with disabilities.

BF is currently finishing a post-doctoral position in the Balance Disorders Laboratory of Dr Faye Horak at Oregon Health Sciences University. He has accepted a faculty position at OHSU and will begin there next year. His main balance expertise is in the domain of Multiple Sclerosis and aging population.
